# Ovarian cancer management in an ESGO ovarian cancer center of excellence: a systematic case study of the interprofessional and interdisciplinary interaction

**DOI:** 10.1007/s00404-023-07368-z

**Published:** 2024-03-20

**Authors:** David J. Krankenberg, Mustafa Zelal Muallem, Klaus Pietzner, Radoslav Chekerov, Robert Armbrust, Carmen Beteta, Wenzel Schöning, Marlene Lee, Julia Klews, Jalid Sehouli

**Affiliations:** 1 Department of Gynecology with Center for Oncological Surgery, European Competence Center for Ovarian Cancer Campus Virchow Klinikum and Charité Comprehensive Cancer Center (CCCC), Medical University of Berlin, Augustenburger Platz 1, 13353 Berlin, Germany; 2 Departement of the Surgical Clinic, Campus Charité Mitte and Campus Virchow-Clinic, Charité – University Clinic, Berlin, Germany; 3 Corporate Member of Free University of Berlin and Humboldt-University of Berlin, Berlin, Germany

**Keywords:** Quality indicators, Patient tracking, Multidisciplinary, Holistic, Contacts

## Abstract

**Purpose:**

With growing knowledge about ovarian cancer over the last decades, diagnosis, evaluation and treatment of ovarian cancer patients have become highly specialized, and an individually adapted approach should be made in each woman by interdisciplinary cooperation. The present study aims to show the variety and extent of medical specialties involved at our institution according to the European Society of Gynecologic Oncology (ESGO) Quality indicators (QI).

**Methods:**

A woman, diagnosed with high-grade ovarian cancer, International Federation of Gynecology and Obstetrics (FIGO) class IVb was selected for a single case observational study. The observation period (total = 22d) comprised preoperative diagnostic procedures, including imaging, the in-patient stay for cytoreductive surgery, and the postoperative course and case discussion at our interdisciplinary tumor board. Data were obtained by self-reporting and by patient file review.

**Results:**

Patient tracking demonstrated an interdisciplinary cooperation of 12 medical specialties [62 physicians (63% male, 37% female)], 8 different types of nursing staff [*n* = 59 (22% male, 78% female)], and 9 different types of perioperative/administrative staff (*n* = 23; male 17,4%, female *n* = 19, 82,6%). Contact with the patient was direct (*n* = 199; 76%) or without face-to-face interaction (*n* = 63; 24%).

**Conclusion:**

The present study demonstrates the high diversity of physicians and the affiliated medical staff, as well as interdisciplinary intersections within teams of a specialized hospital. Matching the ESGO QIs, this report underlines the requirement of an adequate infrastructure for the complex management of advanced ovarian cancer patients.

Future prospective studies are warranted to evaluate the specific procedures and actions to optimize the interprofessional and interdisciplinary workflows.

## What does this study adds to the clinical work?

**Table:** 

This study effectively elucidated the dynamics of personnel resource utilization and allocation within an accredited ESGO ovarian cancer center of excellence across the patient journey. The findings significantly enhance comprehension of the operational intricacies of multidisciplinary teams and their interdisciplinary interfaces. Consideration of studies of this nature is imperative when striving to enhance both economic and patient-related outcomes.	

## Background

Ovarian cancer is placed as the fifth most common cancer in women with over 7300 annual diagnoses and 5326 deaths in Germany [1]. Ovarian cancer makes up a third of all gynecological malignancies and leads to 50% of deaths in this group [2]. As knowledge about ovarian cancer continues to expand, and considering the increasing complexity of this diseas, it is advisable to direct patients toward specialized cancer centers. These centers offer comprehensive surgical expertise, thorough perioperative management, and appropriate adjuvant treatment options. Specialization and experience are considered to be quality indicators for beneficial clinical outcomes in primary and recurrent ovarian cancers [3, 4].

Specialized cancer centers are characterized by a high case volume, the presence of oncologic gynecologists in a multidisciplinary team, regular tumor boards, and participation in clinical trials [3, 5–7].

So far, most studies have evaluated the effectiveness of multidisciplinary teams in specialized centers in comparison to regional or community hospitals regarding overall survival and the chance of complete gross tumor resection.

This study aims to assess the variety and number of medical specialties and healthcare professionals involved in diagnosis, treatment, and postoperative period of a single woman with advanced primary ovarian cancer. In this context, we additionally want to discuss the development of specialized centers the therapeutic benefit and demonstrate the cost-effectiveness of multidisciplinary treatment algorithms.

## Methods

For this single case observational study, a 61-year-old woman with primary ovarian high-grade serous ovarian cancer FIGO IVB was selected.

The patient was referred to our institution by the attending gynecologist because of an increase in abdominal circumference, hypogastric pain, elevated levels of CA125, and a cystic-solid finding of the ovaries, revealed on vaginal ultrasound. Subsequent imaging showed a large pelvic mass with omental caking, peritoneal carcinomatosis, and malignant pelvic, paraaortic, mesenterial, and paracardial lymphadenopathy. She had no pre-existing illnesses with a Karnofsky- and ECOG status of 90% and 0, respectively. The time interval between primary admission and surgery was 9 days.

The patient gave informed consent for the study before being enrolled. The observation period (*n* = 22d) comprised preliminary evaluation, outpatient imaging, and the in-patient stay for cytoreductive surgery and ended with the postoperative case discussion at our interdisciplinary tumor board. The case was discussed once in the pre- and once in the postoperative interdisciplinary tumor conference.

The preoperative tumor conference is composed of gynecologists, radiologists, and visceral surgeons to review the imaging, rule out distant or parenchymatous metastasis, determine possible required bowel resection, and again confirm the indication for surgery. The postoperative tumor conference then comprises gynecologists, pathologists, oncologists, and radiologist and gives a therapeutic recommendation based on the histological specimen.

Additionally, the patient’s case was presented twice in our social conference. The social conference addresses issues like psychological coping with the disease, dietary deficits, improvement of the physical status, and facilitated organization of home care and support. The conference involves nutritionists, physiotherapists, psycho-oncologists, the outreach service, and palliative care team.

For primary cytoreductive surgery, we performed a median laparotomy with en-bloc resection of the uterus, the adnexa, and a peritonectomy of the pelvic- and bladder peritoneum. Because of diffuse tumor infiltration, a sigmoidectomy with an end-to-end descendorectostomy and ileocecal resection followed by a side-to-side ileoascendostomy had to be performed. Furthermore, pelvic, interaortocaval, and suprarenal bulky nodes were resected. Subsequently, the diaphragm was opened to resect a paracardial lymph-node metastasis. The surgery was terminated with a complete tumor resection with no residual macroscopic disease. The final histologic report revealed a high-grade serous ovarian cancer pT3b pN1b (29/50) G3 L1 V0 Pn0 and a low-grade appendiceal mucinous neoplasm pTis pN0 (0/2) R0 in the ileocecal resectate that needed no further therapy. Postoperative medication was comprised of non-steroidal anti-inflammatory drugs, opioids, diuretics, proton pump inhibitors, and antiemetic drugs. Genetic testing did not show a germline mutation in BRCA/HRDgenes.

Data were collected at the clinic for gynecological oncology at the Charité Virchow Campus, an accredited ESGO ovarian and endometrial cancer center of excellence, which makes part of the Comprehensive Cancer Center Charité (CCCC), which is an institution of different medical departments that set the goal to enhance the development of   a precise and tailored cancer treatment through ongoing clinical trials, basic research, and coordination of overlapping different medical specialties for interdisciplinary cooperation.

For efficient tracking, the patient was asked to maintain a list for each day and to write down all individuals she encountered by the day. In addition, all departments (i.e., operating theater, intensive care unit, etc.) were visited individually to generate information about the operating schedules and rotations of the medical staff and healthcare professionals. Furthermore, indirect contacts were ascertained by closely monitoring of the patient´s file deposited in the hospital management software SAP® (Walldorf, Germany) computer program. Direct contacts were defined as face-to-face contact between the patient and the attending physician or medical staff. Indirect contacts were defined as actions as part of the diagnostic or therapeutic algorithm without a face-to-face interaction but directed toward the patient (i.e., pathologists, radiologists, etc.).

Data were analyzed using descriptive statistics.

**Fig. 1:**
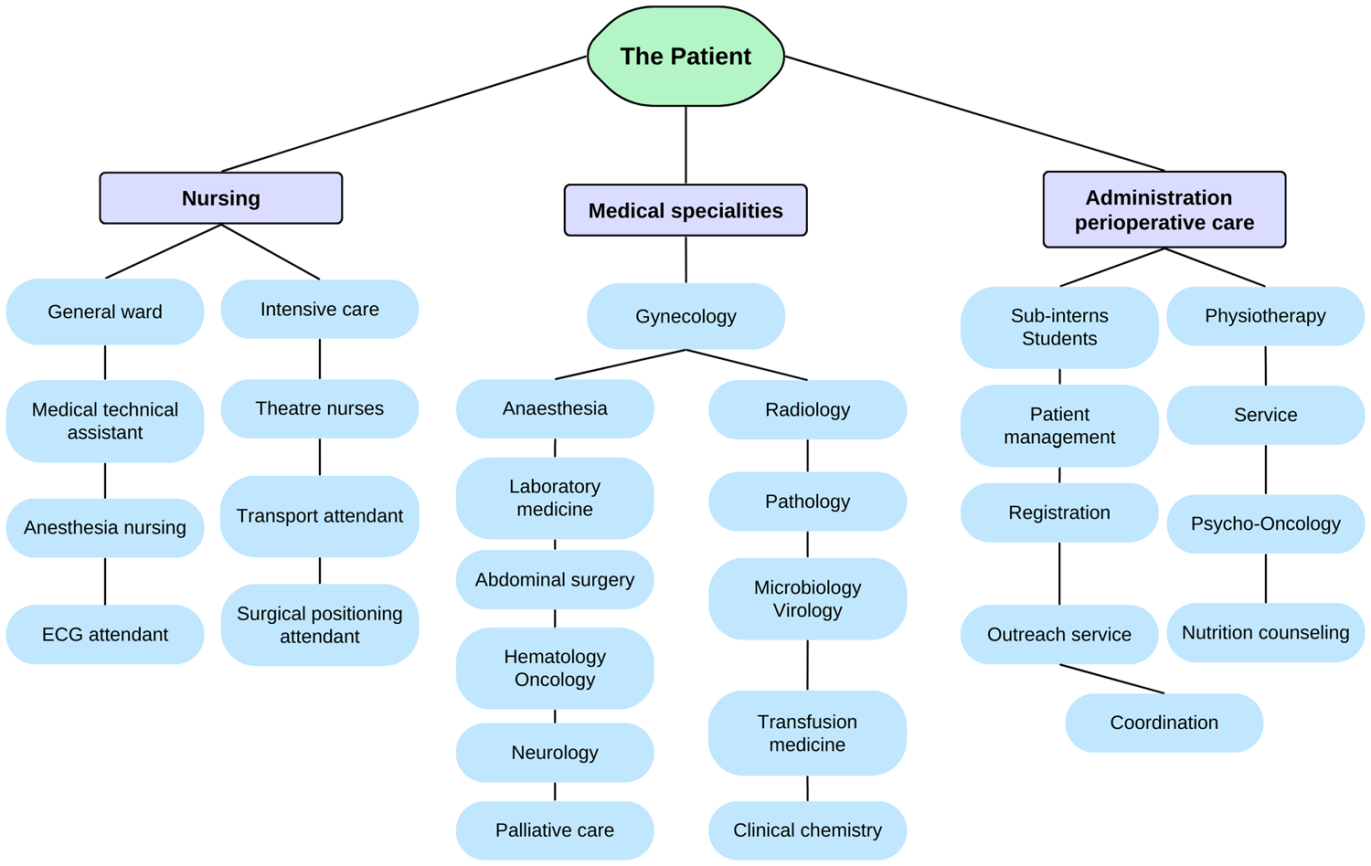
Overview of the multidisciplinary cluster representing all parties involved in the treatment of a single patient. Each segment in descending order respective their occurrence. A total of *n* = 144 individuals were involved

Figure 1 was created using Canva © 2023.

**Fig. 2:**
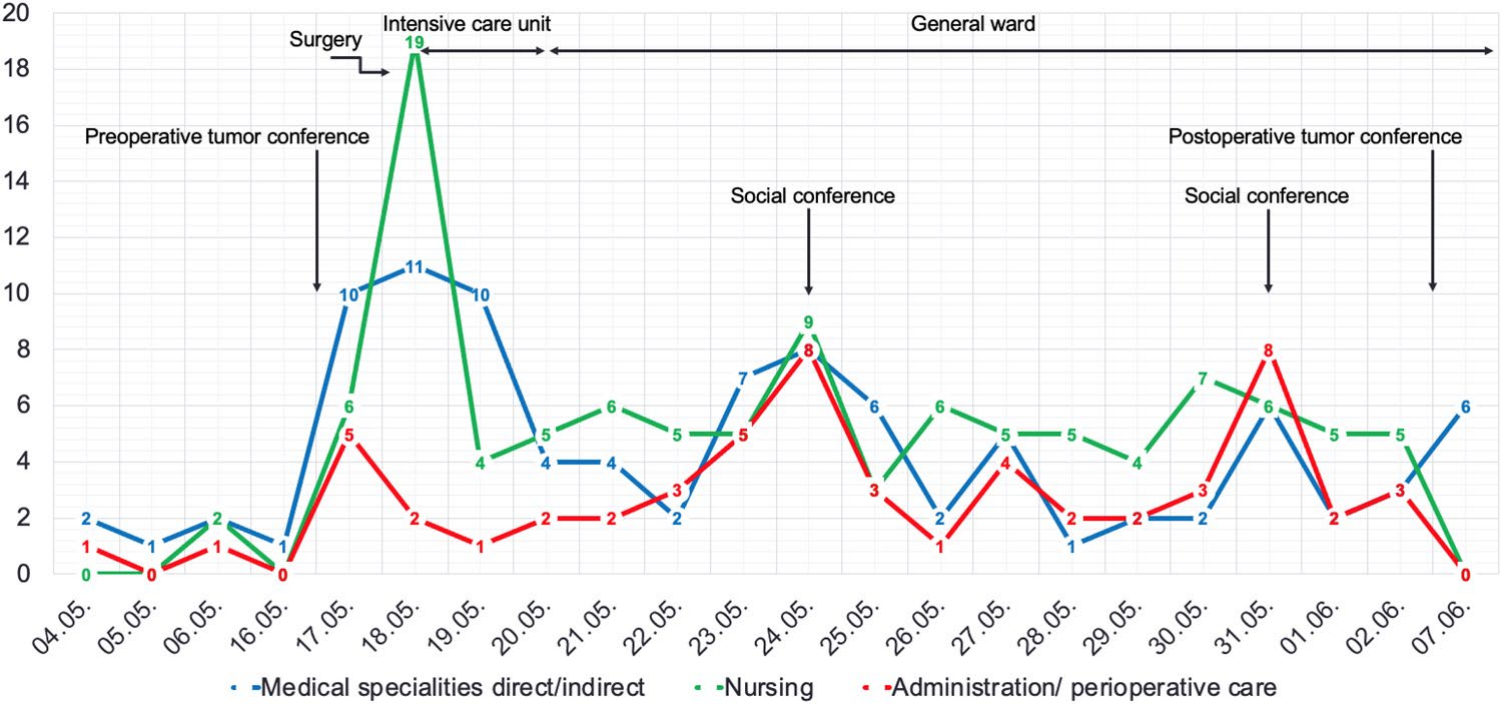
Time course of events, multidisciplinary meetings and healthcare professionals involved

**Fig. 3:**
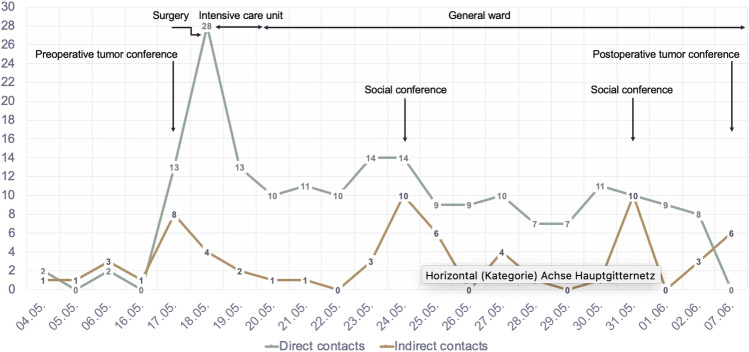
Time course of events, multidisciplinary meetings in the context of direct and indirect contacts

Figures 2 and 3 were created with Windows® Excel 2023 Version 16.75.

## Results

During the observation period, a total of *n* = 144 individuals involved (male *n* = 56; 38,9%, female *n* = 88; 61,1%). Direct contacts represented *n* = 199 (76%; mean* n* = 9,04/d) and *n* = 63 indirect contacts (24%; mean * n* = 2,86/d) with a total number of *n* = 262 patient-oriented contacts. Multiple contacts by the same person within the same day were not considered. The time of hospitalization was 17 days in total. This included the day of admission, the day of the surgery, 1 night of surveillance in the intensive care unit, 1 night at the post-anesthesia care unit (PACU), and 13 days in the general ward. In the postoperative period (*n* = 15), the patient was attended by an oncologic gynecologist 10 times (1.5 times per day), a consultant 8 times, and a resident 7 times (Tables 1, 2, and 3).

**Table 1:** Allocation of the different medical specialties (numbers are rounded to zero decimal places a can differ from 100%)

Medical specialties	Count	% male	% female
1. Gynecology	27% (*n* = 18)	50% (*n* = 9)	50% (*n* = 9)
2. Anesthesia	25% (*n* = 16)	53% (*n* = 9)	47% (*n* = 7)
3. Radiology	12% (*n* = 7)	86% (*n* = 6)	14% (*n* = 1)
4. Laboratory medicine	12% (*n* = 7)	71% (*n* = 5)	29% (*n* = 2)
5. Pathology	7% (*n* = 4)	75% (*n* = 3)	25% (*n* = 1)
6. Microbiology/virology	5% (*n* = 3)	67% (*n* = 2)	33% (*n* = 1)
7. Visceral surgery	3% (*n* = 2)	100% (*n* = 2)	0% (*n* = 0)
8. Neurology	2% (*n* = 1)	100% (*n* = 1)	0% (*n* = 0)
9. Transfusion medicine	2% (*n* = 1)	100% (*n* = 1)	0% (*n* = 0)
10. Palliative care	2% (*n* = 1)	100% (*n* = 1)	0% (*n* = 0)
11. Hematology	2% (*n* = 1)	0% (*n* = 0)	100% (*n* = 1)
12. Clinical chemistry	2% (*n* = 1)	0% (*n* = 0)	100% (*n* = 1)
**Total**	**100% (*n* = 62)**	**63% (*n* = 39)**	**37% (*n* = 23)**

**Table 2:** Allocation of the nursing staff (numbers are rounded to zero decimal places a can differ from 100%)

Nursing	Count	% male	% female
1. General ward	43% (*n* = 26)	0% (*n* = 0)	100% (*n* = 26)
2. Intensive care	19% (*n* = 11)	46% (*n* = 5)	55% (*n* = 6)
3. Medical technical assistant	12% (*n* = 7)	0% (*n* = 0)	100% (*n* = 7)
4. Theater nurses	10% (*n* = 6)	33% (*n* = 2)	67% (*n* = 4)
5. Anesthesia nurses	7% (*n* = 4)	25% (*n* = 1)	75% (*n* = 3)
6. Transport attendants	5% (*n* = 3)	100% (*n* = 3)	0% (*n* = 0)
7. ECG attendants	2% (*n* = 1)	100% (*n* = 1)	0% (*n* = 0)
8. Surgical positioning attendant	2% (*n* = 1)	100% (*n* = 1)	0% (*n* = 0)
**Total**	**100% (*n* = 59)**	**22% (*n* = 13)**	**78% (*n* = 46)**

*ECG* electrocardiogram

**Table 3:** Allocation of the administrative staff and perioperative care (numbers are rounded to zero decimal places a can differ from 100%)

Administration/perioperative care	Count	% male	%female
1. Sub-intern/students	22% (*n* = 5)	40% (*n* = 2)	60% (*n* = 3)
2. Physiotherapy	17% (*n* = 4)	0% (*n* = 0)	100% (*n* = 4)
3. Patient management	13% (*n* = 3)	33% (*n* = 1)	66% (*n* = 2)
4. Service	13% (*n* = 3)	0% (*n* = 0)	100% (*n* = 3)
5. Registration	9% (*n* = 2)	0% (*n* = 0)	100% (*n* = 2)
6. Psycho-oncology	9% (*n* = 2)	0% (*n* = 0)	100% (*n* = 2)
7. Outreach service	9% (*n* = 2)	0% (*n* = 0)	100% (*n* = 2)
8. Nutrition counseling	4% (*n* = 1)	0% (*n* = 0)	100% (*n* = 1)
9. Coordination	4% (*n* = 1)	100% (*n* = 1)	0% (*n* = 0)
**Total**	**100% (*n* = 23)**	**17% (*n* = 4)**	**83% (*n* = 19)**

The postoperative pain service, responsible for adjusting peridural anesthesia (PDA) and managing oral pain medication after removal of the PDA, visited the patient 9 times. Additionally, as part of an accelerated rehabilitation program, a physiotherapist visited the patient 7 times.

The extended duration of hospitalization was attributed to sensory disturbances in the left inner femoral region, prompting the performance of a pelvic MRI to investigate and exclude neural damage. However, the MRI did not reveal any specific findings. Symptoms subsequently resolved prior to the patient’s discharge from the hospital by the intervention of physiotherapists. Decision-making and planning of the surgery were done through interdisciplinary cooperation in the preoperative tumor board.

Our data displays the hospital personnel that comprisess a multidisciplinary team, depicting the standard for patients with gynecological malignancies at our institution-an ESGO Ovarian Cancer Center of Excellence with *n* ≥ 100 cytoreductive surgeries per year. The surgery was performed by a trained oncologic gynecologist, and complete macroscopic tumor resection was achieved.

While gynecology represents the most frequently encountered medical specialty, accounting for 27% of all individuals involved, the anesthesiology department closely follows with a representation of 25%. Visceral surgeons participated in the preoperative tumor conference to assess upper abdominal tumor involvement and determine the potential need for bowel resection. During the surgery, visceral surgeons were called in for a sigmoidectomy, accompanied by an end-to-end descendorectostomy and ileocecal resection. This was followed by the establishment of a side-to-side ileoascendostomy. Radiologists were only consulted during preoperative staging and tumor conference and once for an MRI because of neurological complaints. Patient-oriented contacts by radiologists were indirect. Laboratory medicine, biology/virology, transfusion medicine, and clinical chemistry were involved because of the various blood taking, corona-testing, and preparation of banked blood. Laboratory medicine was occupied with the various Corona testing that had to be carried out every 2 days at that time. Evaluation of blood samples was done by the department of clinical chemistry. Colleagues from neurology were met for consultation but can be exchanged for any other discipline depending on perioperative complications. Colleagues from hematology and oncology, pathology, and palliative care were solely consulted in multidisciplinary team meetings.

Patient-oriented contacts for the preceding disciplines were indirect. Service refers to the service professional distributing meals and beverages, while social service  refers to the support and assistance in custodial measures in the convalescence of the patient.

Additionally, the patient was visited 5 times by a psycho-oncologist, 9 times by a physiotherapist, and was presented 2 times to a nutritionist. Figure 2 effectively presents a peak in the number of involved individuals multidisciplinary meeting days.

After the release from the hospital, a recommendation for 6 cycles of carboplatin AUC5 and paclitaxel 175 mg/KOFm^2^ together with bevacizumab 15 mg/Kg q21d as a first-line systemic therapy was made. Adjuvant and maintenance therapy were administered at the gynecologic outpatient chemotherapy clinic at the Charité Virchow Clinic.

The results aim to quantify the term “multidisciplinary team” rather than evaluate its effectiveness.

## Discussion

The ESGO has developed a list of 10 Qis for advanced ovarian cancer surgery that we implemented in our treatment algorithm. QI 1–3 are related to the caseload in the center, training, skills, and experience of the surgeon and the surgical team. These Qis were covered by trained oncologic gynecologists and general surgeons accordingly, as seen in the results. QIs 4–6 address the overall management of ovarian cancer and their participation in novel therapies and clinical trials as well as the decision-making process in a multidisciplinary team made up of an oncologic gynecologist, radiologists, pathologists with a special interest in gynecologic cancers for interdisciplinary planning before surgery or chemotherapy. Meetings for interdisciplinary dialog and discussion of the case took place twice in the context of the tumor conference and twice as part of the social conference. The value of appropriate anesthesiologic and perioperative care is thematized in QI 7 to ensure low perioperative morbidity and complications. The importance of this QI is reflected by our data as anesthesiology has the second most individuals involved with *n* = 16. We tie this to the daily postoperative visits for pain service and frequent changing anesthesiologists on duty. Furthermore, QI 7 addresses perioperative management, such as, i.e. dietary counseling, pain management, etc., with daily visits and two social conferences for organizing, which can be seen in Figs. 1 and 2. QI 8–10 emphasizes the necessity for complete and transparent interdisciplinary information flow between healthcare professionals for an improved prospective assessment and management of future cases [3]. Palliative care was incorporated as a consistent element among the consulting participants during the social conference, particularly in an initial tumor stage FIGO IVb. Given the exclusive focus on curative therapeutic approaches, involvement was limited to a case discussion accordingly.

Since the nursing staff plays an important role in a patient's the convalescence, we tried to give an appropriate representation of the distribution and diversity of the nursing stuff, as they represent 41% of all individuals involved and are commonly not mentioned in studies addressing multidisciplinary teams. While the larger proportion the nursing staff was occupied with direct patient care, medical technical assistants, ECG attendants, and transport attendants had a logistic and organizational area of responsibility.

New diagnostic tools, molecular and immune histologic subtyping, and consideration of patient- related variables have led to the comprehension that one’s medical specialty does not prove to be sufficient to adequately address the full spectrum of the patient’s needs.

Therefore, as previously stated, the recommended approach for patients with primary and recurrent ovarian cancer involves admission to a specialized center equipped with a multidisciplinary team and a patient-centered treatment algorithm [8, 9].

In Germany, the Federal Ministry of Health initiated the National Cancer Plan in 2008 with the objective to enhance early cancer detection and optimize the infrastructure for oncologic treatment. This initiative encompasses the development of evidence-based treatment guidelines, establishing relevant quality indicators, certification of oncologic centers with a specific emphasis on implementing these guidelines, and documentating the course and therapy in a clinical cancer registry [10]. The German Cancer Society (DGK) has incorporated specific criteria into its audit submission for the certification of gynecologic cancer centers. These criteria underscore the importance of weekly interdisciplinary and multidisciplinary tumor conferences, ongoing medical education, participation in clinical studies, quality management practices, and the provision of patient-tailored medical and holistic approaches. The emphasis is on a team centered around an oncologic gynecologist with a minimum of ≥50 cases involving primary borderline tumors, ovarian cancer, or serous tubal intraepithelial carcinoma (STIC) admitted for surgical intervention [11].

The requirements outlined by the European Cancer Center Programme, formulated by the German Group on Gynecological Oncology (AGO), align with and are equivalent to the German requirements [12].

The concept of multidisciplinary teams and interdisciplinary collaboration has not always been the prevailing standard of care. The development of the currently embraced multidisciplinary approach originated with a linear treatment model, wherein each discipline addressed the patient’s case from its perspective. This method involved transferring the patient sequentially from one discipline to another, leading to a lack of feedback and uncontested decision-making [13].

The unchallenged linear approach can have detrimental effects, particularly in specialized disciplines, where a high level of specialization may result in fragmentation when interdisciplinary dialog is not ensured [14].

The patient-centered care was then the next concept after the linear approach and signifies the continuous circular information exchange between medical disciplines around the patient, resulting in a convergence of competencies of multiple disciplines beyond one´s knowledge. This approach leads to diagnostic and therapeutic opportunities that are continuously received and shared between the different specialties [13, 15].

However, the now accepted multidisciplinary concept, combines the patient-centered approach and adds another layer. Since the quality of life has become an equally important issue in cancer care, the additional layer comprises services like psycho-oncology, physiotherapy, nutrition counseling, outreach service, etc., not only medical but holistic, as the quality of life should be considered one of the major therapeutic endpoints of tailored medicine [13].

Today, the centerpiece of multidisciplinary teams in gynecologic cancer centers is formed by oncologic gynecologists, who perform primary and recurrent debulking surgeries and decide on systemic therapy [6].

Vernooij et al. showed a positive surgical outcome, especially in advanced ovarian cancers with stage III or higher, when done by an oncologic gynecologist [6].

Patients with advanced ovarian cancer and no gross residual disease (*R* = 0) have the best prognosis as survival is inversely correlated with the volume of residual disease [16].

Centers with a higher caseload and expertise lead to a significantly lower hazard of death when surgery is done by experienced oncologic gynecologists in comparison to gynecologists in peripheral centers [17].

This was again confirmed by Woo et al., who evaluated institutions with an oncologic gynecologists on site as opposed to the community or general hospital by meta-analysis of three studies [18–20] with a total of 9041 women and found that the survival was better in specialized centers, as opposed to community or general hospitals (comparing risk of death among women treated in specialized centers with that among women treated in non-specialized centers: HR 0.90; 95% CI 0.82–0.99). Furthermore, the difference was evaluated between teaching and regional cancer centers vs. community or general hospitals (HR 0.91; 95% CI 0.84–0.99) by a meta-analysis of another three studies [18, 21, 22].

Outside the meta-analyses, the studies individually did not find a significant difference between the hospital settings. The study's results are at risk to be biased because of retrospective data and pooled estimates [23].

Despite the general heterogeneity in study results, cumulative data suggest that centralized and teaching centers provide a better result in comparison to regional or general hospitals [24].

The current advancements in clinical standards, scientific research, therapeutic outcomes, and economic considerations exert pressure on clinical organizations, physicians, and healthcare professionals. This dynamic presents challenges in the context of the multidisciplinary approach, as there is a need to balance the beneficial effects of such an approach and the associated costs. Finding a midpoint that meets the requirements for both effectiveness and economic feasibility becomes a critical consideration [25]. Regarding the cost-effectiveness of ovarian cancer treatment in specialized and non-specialized clinics, Bristow et al. demonstrated that patients treated in expert centers had an overall cost per patient of 50,652$ and effectiveness of 5.12 quality-adjusted life years (QALYs) with a 75% rate of optimal primary cytoreductive surgery in comparison to 39,957$ per patient and an effectiveness of only 2,33 QALYs and a 25% of optimal primary cytoreductive surgery in less-experienced center. The expert center, however, showed a cost-effectiveness of 9893$ per QALY, while the less-experienced center had a cost-effectiveness of 17,149$ per QALY [26].

With regard to the aforementioned, we believe that assessments of this nature, which provide an overview of personnel resources and their interfaces, can serve as the foundation for discussions aimed at enhancing the infrastructural and economic framework within today’s ovarian cancer management. The objective is to influence public policies with the dual goals of reducing expenditures in centralized centers while simultaneously improving medical outcomes and fostering interprofessional dialog.

Due to methodological limitations, the generalizability of our observations to all other ESGO centers is constrained, given the anticipated high heterogeneity in individual infrastructure and patient characteristics. Nevertheless, we believe these results provide relevant information for the clinical routine and can be used as a basis for future scientific initiatives.

### Strengths and weaknesses

The value of contacts can be arbitrary as the number of involved individuals and contacts do not coercively correlate with the impact the individual had on the patient, as communicated contents were not monitored.

Subjective perception of the patients during patient tracking and overall therapeutic benefit were not evaluated, and findings in this case study might not be representative for a larger cohort. Hence, it is inconclusive whether reducing the number of physicians and healthcare professionals would yield enhanced continuity of care for the patient, economic effectiveness, and improved medical outcomes. Multiple contacts of the same persons within 1 day were not tracked, and we are aware that results cannot be translated to all ovarian cancer patients.

To conclude, multidisciplinary and interdisciplinary often seem to be used in the same sense, whereas we believe that the term multidisciplinary merely signifies the presence of different disciplines. This case study shows the vast number of healthcare professionals involved in the treatment algorithm according to the QIs of the ESGO for patients with advanced ovarian cancer.

This report underlines the requirement for an adequate infrastructure for the complex management of advanced ovarian cancer patients. Future prospective studies are warranted to evaluate the specific procedures and actions to optimize the interprofessional and interdisciplinary workflows. The preoperative period and the subsequent cancer therapies should be integrated in this context.

## Data Availability

The data that support the findings of this study are available from the corresponding author upon reasonable request.
